# Laparoscopic Management of an Intrauterine Device Perforating the Uterus and Adherent to the Rectum and Ovary: A Case Report

**DOI:** 10.7759/cureus.101683

**Published:** 2026-01-16

**Authors:** Maya N Fakih, Zaid R Al-Wahab

**Affiliations:** 1 Undergraduate Admissions, University of Michigan, Ann Arbor, USA; 2 Obstetrics and Gynecology, Corewell Health William Beaumont University Hospital, Royal Oak, USA

**Keywords:** bowel adhesion, intrauterine device, iud perforation, laparoscopy, robotic surgery

## Abstract

Management of intrauterine device (IUD) perforation can be complex when adjacent pelvic organs are involved. Laparoscopic removal is commonly performed; however, cases involving bowel adherence may require intraoperative assistance from additional surgical services. We report the case of a 31-year-old gravida 2, para 2 (G2P2) woman with a history of cesarean delivery who presented with pelvic pain five weeks postpartum and was found to have an IUD perforating the posterior uterine wall, with adherence to the rectum and left ovary. The patient underwent robotic-assisted laparoscopic removal of the IUD. Gynecologic oncology was consulted intraoperatively due to availability and expertise in pelvic dissection to assist with the removal of the IUD from the colon, in collaboration with colorectal surgery. The device was removed intact; associated uterine and rectal defects were repaired, and hemostasis was achieved. The postoperative course was uncomplicated, with complete resolution of symptoms. Published literature describing robotic-assisted approaches for complex IUD perforation involving adjacent pelvic organs remains limited. This case contributes descriptive clinical experience regarding multidisciplinary, robotic-assisted management of complicated IUD migration.

## Introduction

Uterine perforation associated with intrauterine device (IUD) use has an incidence of approximately 1 in 1,000 insertions [1]. The increasing use of IUDs has inevitably led to more patients presenting with complications. Although perforation remains rare, it can be serious and requires prompt recognition and management, particularly when adjacent structures such as the bowel or rectum are involved [2-4]. Removal of a partially perforated IUD depends on the compartments involved. Devices primarily located within the uterine cavity can usually be removed more easily, whereas those embedded deeper within the myometrium or extending into the peritoneal cavity may require laparoscopic removal.

A type A perforation involves a device mainly situated in the uterine cavity (A1) or primarily within the myometrium (A2). Devices classified as A1 are generally easier to remove than those classified as A2, as removal of myometrially embedded devices can be difficult or hazardous. In type B perforations, the IUD lies entirely within the myometrium and cannot be visualized by hysteroscopy or laparoscopy. In type C perforations, the device protrudes into the peritoneal cavity while remaining fixed in the myometrium. Type D perforations involve extension of the device across all three compartments. In D1 perforations, the device extends into the peritoneal cavity while remaining partially embedded in the myometrium, whereas D2 perforations involve deeper extrauterine extension with greater involvement of surrounding structures, making surgical removal more complex.

Type D perforations require detailed preoperative assessment to guide surgical planning, as deeper extensions such as D2 are more challenging to remove laparoscopically. In cases of complete perforation, the device may migrate into the pouch of Douglas or adhere to the omentum, occasionally necessitating excision of attached tissue. Extreme care is required during removal, as forceful extraction can result in tissue injury and hemorrhage [1]. Invasion of surrounding structures is uncommon, occurring in approximately 15% of uterine perforations, with intestinal involvement most often affecting the sigmoid colon and less commonly the small intestine or rectum (each approximately 21%) [5].

This case report describes a 31-year-old gravida 2, para 2 (G2P2) woman with an IUD that perforated through the uterus and into the bowel. Intraoperatively, the uterus, fallopian tubes, and ovaries were grossly normal; however, the rectum and colon were adherent to the left posterior uterine wall, and the left ovary was also adherent posteriorly. The IUD was identified perforating through the uterus and lying adjacent to the bowel, without evidence of stool spillage. The device was removed, the uterine perforation was repaired with Vicryl sutures, and hemostasis was achieved using Vistaseal. The patient received intraoperative antibiotics and had an uncomplicated postoperative recovery.

## Case presentation

The patient was a G2P2 woman who was breastfeeding and had an IUD inserted approximately two years prior to presentation, at five weeks postpartum following an uncomplicated repeat low transverse cesarean section. The IUD insertion was documented as uncomplicated. The patient presented for an IUD check at an office visit six weeks after insertion and reported pain near the rectum following IUD placement, as well as pain with bowel movements during the first two weeks after insertion.

She reported bleeding only during the first week after insertion, with no further abnormal bleeding or persistent pain thereafter. On physical examination at that visit, the IUD strings were visualized at the cervix and appeared to be of normal length. The patient was not seen again for IUD-related complaints at that time.

Approximately 19 months after IUD insertion, the patient reported a recurrence of mild pelvic pain, which subsequently worsened following a fall. She presented for further evaluation approximately two years after IUD insertion. She stated that the pain became significantly worse after the fall and was localized to the lower abdomen. She again recalled experiencing pain and cramping during the first week following IUD insertion. After the fall, the pain felt different from her usual menstrual discomfort, and she began experiencing increasingly painful menses with delayed cycles. She denied any prior history of irregular menstrual cycles. The pain worsened with carrying heavy items and was associated with a foul odor and vaginal discharge. Physical examination at that time was unremarkable, and the IUD strings were again visualized at the cervix with appropriate length.

**Figure 1:**
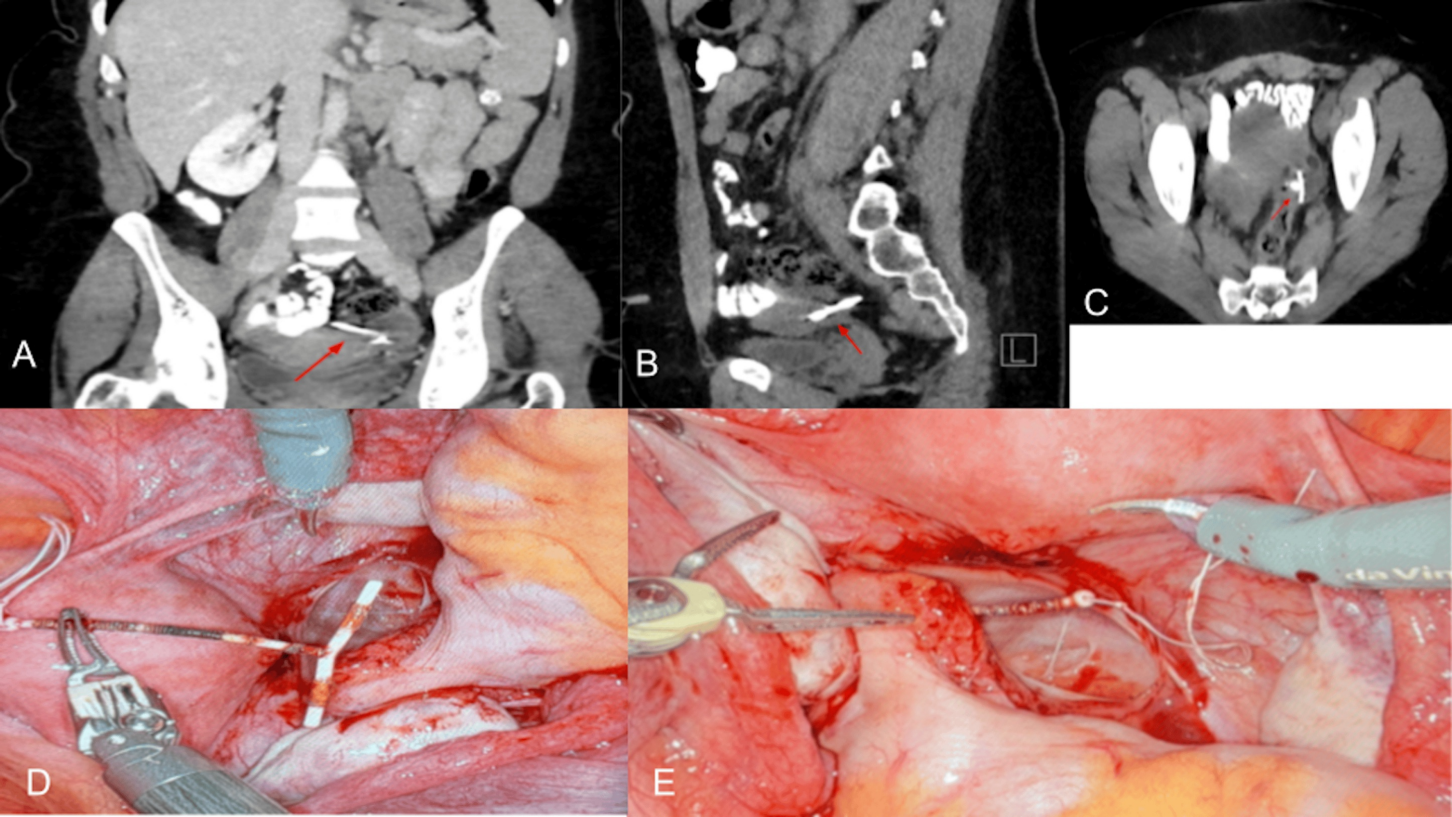
(A) Coronal (frontal plane), (B) sagittal, and (C) axial CT scan showing misplaced intrauterine device (IUD) within the pelvis, positioned posterior to the uterus and adjacent to the rectum. (D, E) Intraoperative view demonstrating the extraction of an intrauterine device (IUD) during robotic-assisted pelvic surgery.

The IUD was not identified within the uterine canal; however, the IUD strings remained visible at the cervix. The remainder of the device was visualized transversely in the left adnexa, posterior to the uterus, with possible involvement of the bowel. A computed tomography (CT) scan of the abdomen and pelvis was therefore obtained (Figure 1).

The radiologist reported “a linear metallic density partially projecting within the uterine fundus and predominantly within the left adnexa, which may represent postsurgical changes versus a perforated intrauterine device.” The patient was subsequently scheduled for urgent operative laparoscopy.

The patient was taken to the operating room and placed under general anesthesia for robotic-assisted laparoscopic removal of the IUD. Prophylactic antibiotics were administered, including 2 g cefazolin (Ancef) and 500 mg metronidazole (Flagyl). Pelvic survey revealed the rectosigmoid colon adherent to the posterior uterine wall. The left ovary was also noted to be adherent posteriorly to the uterus. Gynecologic oncology and colorectal surgery were consulted intraoperatively to assist with the evaluation and repair. Adhesiolysis was performed, and the IUD was subsequently identified. The device was carefully removed intact from the sigmoid colon, with strings attached, using an endoscopic retrieval bag. No stool spillage was noted, and there was no gross evidence of bowel lumen penetration.

The rectal wall defect was closed with 3-0 silk sutures and reinforced with 3-0 Vicryl seromuscular sutures. To further evaluate the integrity of the sigmoid colon, an intraoperative air leak (“bubble”) test was performed and was negative. Given the absence of stool spillage and a negative leak test, colonoscopy was deferred. Minimal bleeding was noted from the posterior uterine wall at the perforation site, and hemostasis was achieved with suture ligation (Figure 1).

## Discussion

IUD perforation is a potentially serious complication that occurs in approximately 1 in 1,000 insertions. Although rare, migration of an IUD into adjacent pelvic organs, such as the bowel or ovary, may result in adhesions, infection, and, in some cases, infertility if not managed appropriately. The most common sites of extrauterine IUD migration are the omentum and the sigmoid colon. If left undiagnosed, perforated IUDs may lead to abscess formation, bowel obstruction, or peritonitis. Prompt diagnosis and surgical intervention are therefore critical to reduce long-term morbidity and prevent potentially life-threatening sequelae.

Management of IUD perforation depends on the location of the device, the presence of adhesions, and the patient’s clinical presentation. While some cases may be managed conservatively or with hysteroscopic removal, many require a surgical approach. Laparoscopy is often the treatment of choice for symptomatic extrauterine devices, particularly when adjacent organs are involved. However, as demonstrated in this case, laparoscopy alone may be insufficient without multidisciplinary support. This case highlights an advanced approach using robotic-assisted laparoscopy with intraoperative consultation from both gynecologic oncology and colorectal surgery. Although robotic surgery has been described for other complex pelvic pathologies, its use in IUD perforation with bowel involvement remains limited in the literature. To our knowledge, this is one of the first reported cases of successful robotic-assisted IUD removal in the setting of rectal and ovarian adhesions. These findings suggest that robotic-assisted laparoscopy may be a safe and effective option for complex presentations of IUD perforation and warrant further evaluation in larger clinical studies.

## Conclusions

IUD perforation is a rare but potentially serious complication that can result in adhesion or injury to nearby pelvic organs, such as the bowel and ovary. This case highlights the importance of maintaining a high index of suspicion for IUD migration in patients presenting with persistent pelvic pain, even months after insertion. Prompt imaging evaluation is critical for diagnosis and surgical planning. Successful management relies on a multidisciplinary approach involving gynecology, colorectal surgery, and oncology teams to ensure safe removal and repair of affected structures. In this case, careful adhesiolysis, defect closure, and use of hemostatic agents led to a favorable outcome without complications. Continued awareness, early recognition, and collaborative surgical management are key to minimizing morbidity in patients with IUD perforation involving adjacent organs.
